# The Roles of DNA Demethylases in Triple-Negative Breast Cancer

**DOI:** 10.3390/ph14070628

**Published:** 2021-06-29

**Authors:** Shoghag Panjarian, Jean-Pierre J. Issa

**Affiliations:** Coriell Institute Research Department, Coriell Institute for Medical Research, Camden, NJ 08103, USA; jpissa@coriell.org

**Keywords:** TNBC, TET enzyme, DNA methylation

## Abstract

Triple-negative breast cancers (TNBCs) are very heterogenous, molecularly diverse, and are characterized by a high propensity to relapse or metastasize. Clinically, TNBC remains a diagnosis of exclusion by the lack of hormone receptors (Estrogen Receptor (ER) and Progesterone Receptor (PR)) as well as the absence of overexpression and/or amplification of HER2. DNA methylation plays an important role in breast cancer carcinogenesis and TNBCs have a distinct DNA methylation profile characterized by marked hypomethylation and lower gains of methylations compared to all other subtypes. DNA methylation is regulated by the balance of DNA methylases (DNMTs) and DNA demethylases (TETs). Here, we review the roles of TETs as context-dependent tumor-suppressor genes and/or oncogenes in solid tumors, and we discuss the current understandings of the oncogenic role of TET1 and its therapeutic implications in TNBCs.

## 1. Triple-Negative Breast Cancer

Triple-negative breast cancer (TNBC) is a heterogenous disease that is genetically complex and is defined by lack of estrogen (ER)- and progesterone (PR)-receptor expression and the absence of overexpression and/or amplification of human epidermal growth factor receptor 2 (HER2). TNBC constitutes 15–20% of all breast cancer subtypes. It is more common in African American women, premenopausal women, and in BRCA1/2 mutation carriers. TNBC is also a very aggressive subtype with high tumor grade and high mutation rate and proliferation index compared to other breast cancer types. TNBC has the worst outcome with a relative 5-year survival rate of 91% in localized disease, 65% in locally advanced disease, and 11% in metastatic disease. Patients with TNBC who do not achieve complete pathologic response with chemotherapy and will typically have tumor recurrence or succumb to metastatic disease in less than 5 years of initial diagnosis. Poor outcome in this subtype is also due to lack of available targeted therapies which are commonly used in ER+, HER2+ subtypes [1,2,3].

Twenty years ago, using the microarray technique, four intrinsic breast cancer subtypes were identified (luminal A, luminal B, HER2-enriched, and basal-like). This was one of the first reports on basal-like breast tumors [4]. This subgroup of breast cancer samples was characterized with low expressions of HER2, ER receptor, ER-associated genes, and high basal gene expression profile [4]. However, as molecular techniques advanced, classifications of intrinsic breast cancer subtypes also evolved. Molecular advances such as in omics (genomics, epigenomics, proteomics, and transcriptomics) and mass spectrometry techniques, fostered the subclassification of TNBC from morphological diagnosis to molecular (DNA, RNA, and protein) and immune profiling [5,6]. TNBC subgroups were refined based on transcriptional signature into two different groups. Though there were different number of subtypes in those studies (six in one report and four in the other), currently it is well accepted that there are four distinct subtypes with clinical correlation—luminal-AR (LAR), mesenchymal (MES), basal-like immune-suppressed (BLIS) and basal- like immune-activated (BLIA) [7,8,9]. By this time, technological advances had also enabled identification of epigenomic signatures of breast cancers and expanded understanding of the molecular classification of breast cancers. Epigenetic studies revealed that TNBC patient samples display widespread genome-wide DNA hypomethylation compared to other breast cancer types and to normal breast controls [10,11]. Further attempts to understand the distinct breast cancer subtypes were based on proteomic signatures [12] and by the integrative approach of proteogenomic signatures [13,14].

Although the four TNBC subclassifications are not yet routinely used in the clinical setting, these advances are important steppingstones to making this heterogenous group of cancer more amenable to new therapeutic insights and potentially to personalized medicine. These discoveries also help shift the paradigm from what TNBC is, rather than to what it is not, and possibly to further sub-stratification. In this review we will present the functional role that epigenetic modifiers such as DNA demethylases play in driving this disease and how we envision their therapeutic potential.

## 2. Epigenetics and Breast Cancer

Epigenetics refers to heritable molecular determinants of gene expression in the absence of changes in DNA sequences. The molecular mechanisms contributing to epigenetic changes in gene expression are DNA methylation, histone modifications, and non-coding RNA regulation. These mechanisms are critically important during normal development and their aberrations have been associated with diseases such as cancer. Histone modifications are post-translational modifications (methylation, acetylation, phosphorylation, ubiquitination, glycosylation, and others) of the histone tail residues carried by different enzymes such as histone methyltransferases (HMTs), demethylases (HDMs), acetyltransferases (HATs), and deacetylases (HDACs). These different modifications and the expression levels of the modifying enzymes can influence the open and closed states of the chromatin that lead to changes in gene expression. Many aberrations in these modifications, including both increases or depletions in these marks and their respective modifying enzymes, have been found in breast cancer and are associated with breast cancer initiation, progression, and prognosis [15,16,17,18,19,20]. Non-coding RNAs, such as microRNAs (miRNAs) and long non-coding RNAs (lncRNAs), are untranslated RNA molecules that can also influence gene expression and their expression can also be regulated by epigenetic mechanisms. In breast cancer, some of these ncRNAs are pro-tumorigenic and enhance proliferation invasion and evasion of apoptosis while others function as tumor suppressors [21,22,23,24,25]. Furthermore, they have been used as potential promising biomarkers for subtyping breast cancers, as treatment response and overall survival [26,27]. The third epigenetic mechanism that plays an important functional role in breast cancer biology is DNA methylation. DNA methylation is comprised of three groups of modifiers—writers, readers, and erasers. The writers are enzymes that catalyze the addition of a tag (methyl) that is read and interpreted by the readers (proteins with a methyl-binding domain), while erasers (the focus of this review) play an important role in removing the methyl tag highlighting the importance and the possibility of epigenetic plasticity.

### 2.1. DNA Methyltransferases

DNA methyltransferases (DNMTs) catalyze the transfer of a methyl (CH_3_) group to the 5-position of cytosine when followed by guanosine (CpG) in DNA. Methylation is initiated by de novo methyltransferases DNMT3A and DNMT3B. These enzymes are highly expressed during embryogenesis and are crucial for establishing methylation patterns. The methyl mark is then maintained by DNMT1 which is the most abundant DNMT in adult mammalian cells and functions as a maintenance methyltransferase that faithfully copies and propagates the methyl mark during replication [28,29]. DNA methylation is a stable signal which serves as a regulatory mechanism for gene expression, embryonic development, genomic imprinting, and memory signal [30,31,32]. In normal tissues, most CpG sites are highly methylated while CpG sites that are in CpG-rich regions or CpG islands (CGI), often in promoters, are unmethylated. However, this pattern of DNA methylation changes, at different magnitudes, in normal aging tissues as well as in diseases such as in cancer [33,34,35]. In cancer, including breast cancer, there is genome-wide global hypomethylation and localized hypermethylation. The functional consequences of these DNA-methylation-associated changes in gene expression are context-dependent. For example, promoter CGIs of critical tumor-suppressor genes tend to gain methylation that leads to repression of these genes while highly methylated CpG sites lose methylation globally, while global loss of methylation, such as in the intergenic regions, has a lower effect on gene expression but can lead to genomic instability, activation of endogenous retrotransposons, induction of immune response, and other effects [34,36,37,38]. There are currently two FDA-approved DNMT inhibitors, as shown in Table 1. DNMT inhibitors decitabine and azacytidine are cytosine analogs that incorporate into newly synthesized DNA and form a covalent bond with DNMTs leading to DNMT degradation and genome hypomethylation. These inhibitors have been FDA-approved for myelodysplastic syndromes (MDS) and are recommended for acute myeloid leukemia (AML) patients who cannot tolerate chemotherapy [35,39]. One limitation of decitabine and azacytidine, is their low plasma half-life [40]. Therefore, guadecitabine was developed to be resistant to degradation by cytidine deaminase with a gradual release of decitabine—the active metabolite. Guadecitabine is currently in several phase III clinical trials for AML and MDS [41,42,43,44]. Additionally, there are other non-nucleoside inhibitors of DNMT activity in preclinical models and these have been recently reviewed by Yu J et al. [45].

### 2.2. DNA Demethylases

**Figure 1:**
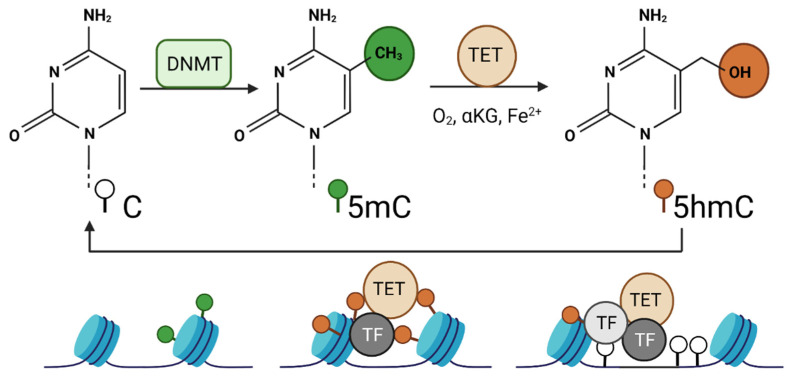
TET-mediated DNA modification and gene expression. Cytosine (*C*) residue is modified by DNMT enzymes into 5-methylcytosine (5mC) which is then oxidized into 5-hydroxymethylcytosine (5hmC) by the enzymatic activity of TETs. TET enzymes depend on oxygen, alpha-ketoglutarate (αKG), and iron (Fe^2+^) for the oxidation reaction. TET enzymes are recruited to the DNA on the nucleosome by transcription factors (TFs). TET enzymes oxidize 5mC to 5hmC and in successive oxidation steps cytosine is unmodified and demethylated. This allows binding of other transcription factors and remodeling of the nucleosome.

Compared to gain of methylation, loss of methylation was only thought of as a passive process due to lack of maintenance during replication. However, in early the 2000s, the discovery of tet-eleven translocation (TET) proteins—DNA demethylases (erasers)—revealed the presence of an active demethylation process in early embryogenesis. TET enzymes were discovered by Rao and colleagues when looking for paralogues of the *Trypanosoma brucei* JBP1 and JBP2 enzymes. In this parasite, these Fe (II)/alpha-ketoglutarate-dependent oxygenase family enzymes are involved in the oxidation of the thymine base in DNA to 5-hydroxymethyluracil [46]. The TET family of proteins include TET1, TET2, and TET3 enzymes. These enzymes (Figure 1) are members of the large super family of 2-oxoglutarate (2OG) and Fe (II)-dependent dioxygenases that convert 5-methylcytosine (5mC) into 5-hydroxymethylcytosine (5hmC) which then can be further oxidized into 5-formylcytosine (5fC) and 5-carboxylcytosine (5caC) [47]. These methylcytosine modifications can be restored to unmodified cytosine through replication-dependent passive dilution. In addition, the highly oxidized derivatives (5fC and 5caC) are excised by thymine DNA glycosylase (TDG), and the base excision repair (BER) pathways regenerate the abasic site with unmodified cytosine leading to demethylation of the 5mC. TET enzymes, though primarily expressed during embryogenesis, can be detected at different levels in different adult cells [46,48,49].

All three TET enzymes harbor a common core catalytic domain comprised of a conserved cysteine-rich domain and a double-stranded beta-helix (DSBH) domain that contains key residues that interact with cofactors and substrates. For the oxidation reaction, TET enzymes require 2OG, Fe (II), and molecular oxygen. The oxidation reaction generates CO_2_ and succinate. Accumulation of succinate and fumarate as well as D-2HG which is generated by mutant isocitrate dehydrogenase (IDH) enzymes, can inhibit the TET enzyme demethylases while vitamin C serves as an essential co-factor. At the amino-terminal region, both TET1 and TET3 contain a CXXC domain that targets the protein to unmethylated CpG islands. TET2 does not contain a CXXC domain but pairs up with IDAX protein, an independent CXXC containing protein [50,51,52,53]. The CXXC domain has been shown to affect the distribution of the TET proteins such that TET1 and it is enriched at the borders of CpG islands and serves as guardian of the unmethylated status while TET2 is enriched in non-CpG islands, enhancers, and gene bodies [54,55,56].

Besides differences in genomic distribution of TET enzymes, they could also interact with different partners and could demethylate independently of their catalytic activity. For example, catalytic activity of TET1 is important in regulating the expression of PGC7 which displaces UHRF1 and impedes DNMT1 recruitment, leading to DNA demethylation [57]. In in vitro studies, overexpression of TET1 enzymes does not induce demethylation in most CpG sites but instead protects the unmethylated sites from methylation [54]. TET1 also interacts with the PRC2 complex and represses lineage-specific genes [58].

### 2.3. DNA Methylation in TNBC

DNA methylation array studies revealed that epigenetic alterations in breast cancer methylome are not only common but can also be used to stratify different breast cancer subtypes, correlate with cancer stage, and predict clinical outcomes [10,59,60]. TNBC tumors are the most hypomethylated compared to other subtypes such as the ER-positive tumors and normal breast controls [10,11]. Moreover, it was shown that the hormone-negative breast tumors characterized by the absence of CpG island methylator phenotype, were associated with high metastatic risk and death [38]. More recently, it was also shown that the hypomethylated TNBC tumors had worse overall survival [61]. Although the hypomethylation was one of the earliest gene-specific DNA methylation abnormalities described in cancer, only recently are its causes being elucidated. In recent years, several research studies pursued the understanding of the mechanisms of hypomethylation in cancer, particularly in hematological cancers. In some cases of acute myelogenous leukemia, hypomethylation could be explained by inactivating mutations in DNMT3A but these mutations are rare in solid tumors and cannot be driving hypomethylation in TNBCs [62,63]. Therefore, TNBCs provide an opportunity to understand the mechanistic basis of DNA hypomethylation, the association between levels of TET enzymes and their functional role in hypomethylation, the consequences of hypomethylation, and the association with prognosis.

## 3. Are TET Enzymes Tumor-Suppressors or Oncogenes?

There is confounding evidence about the functional roles of TET enzymes particularly in solid tumors [64]. In numerous publications, TETs are considered as putative tumor suppressors [65,66,67] and in other reports, TET enzymes are considered to have oncogenic potential [68,69]. These controversies are also reported even in the same cancer type such as in breast cancer [66,70,71]. Are TET enzymes then both tumor suppressor and oncogene depending on cellular context, mode of regulation, different interacting partners, and upstream and downstream signaling pathways?

### 3.1. Tumor Suppressor Activity of TETs

In multiple cancer cell lines and primary tumors, whole genome CpG methylation analysis showed that TET1, but not TET2 or TET3, was downregulated by promoter methylation compared to the normal controls. It is commonly known that DNA methylation alterations, such as global hypomethylation and localized hypermethylation at promoters of tumor-suppressor genes, is a hallmark of cancer [35]. Therefore, gain of methylation at the TET1 promoter may inactivate this gene and may play a significant role in multiple cancers including breast cancer. Indeed, the multiple cancer cell lines studied, including nasopharyngeal, esophageal, lung, gastric, colon, breast, cervical, and renal carcinomas, as well as lymphomas, showed high frequency TET1 promoter methylation and silencing [72]. Furthermore, treatment with DNMT inhibitor 5-aza-dC with or without histone deacetylase (HDAC) inhibitor, reversed the promoter methylation and repression of TET1 in cancer cell lines. Of note, TET1 promoter methylation was also detected in primary tumors for the same cancer types as the cancer cell lines, but less frequently than in cell lines, which emphasizes the importance of microenvironment and cellular context in the regulation of TET1. Furthermore, functionally, ectopic expression of TET1 catalytic domain in cancer cell lines reactivated silenced tumor-suppressor genes (*DLit2*, *ZNF382*, *HOXA9*, and *DKK1*) and significantly suppressed clonogenicity of cancer cells compared to the catalytically dead mutant [72,73]. These findings support a tumor-suppressive role for TET1 which is predominantly silenced by promoter methylation in cancer [72].

In a meta-analysis of 3100 patients, the association between TET1 expression and prognosis of patients with breast, colorectal, cholangiocarcinoma, endometrial, lung, ovarian, gastric, renal, and liver cancer was studied. In the pooled analysis, higher TET1 expression in cancer was associated with better overall survival (OS) and in subgroup analysis higher TET1 expression was associated with better OS in respiratory tumors as well as in breast cancer from Asian patients. In this analysis, there were four breast datasets, three of which were Asian breast datasets. However, in the pooled subgroup analysis, the non-Asian breast dataset, which showed the opposite association between TET1 and OS, was excluded and therefore the prognostic role of TET1 was only established in the Asian dataset [73]. Therefore, the prognostic value of this association between TET1 and overall survival might only hold true in the Asian dataset and should be cautiously inferred and interpreted in the context of other breast cancer datasets and subtypes. The various datasets pooled and compared in this study used different cut-off values and measurements for TET enzyme; some studies used mRNA, others used protein levels. In the future, if a prognostic value of TET enzyme is to be established, there should be a unified method for TET measurement, and studies should be designed to include larger datasets and various tumor types. Several studies demonstrated a positive association between high expression levels of TET1 and better overall survival of breast cancer patients and the opposite was also reported whereby low levels of TET1 was associated with worse overall survival [66,74,75]. These studies attribute the association to the role of TET1 in hypomethylating and therefore reactivating tumor-suppressor genes such as *TP53* and *TIMP*, which in turn would lead to suppression of tumor development, invasion, and therefore better overall survival.

Another study that supports the tumor-suppressive role of TET was shown through the inhibition of alpha ketoglutarate dehydrogenase (KGDH). Inhibition of this enzyme increases the alpha ketoglutarate levels which is a cofactor of TET enzyme in the oxidation reaction. In this study, alpha ketoglutarate increased TET activity in in vivo model of highly aggressive metastatic breast cancer (4T1) and in cell lines. The increase in TET levels was associated with increase in downstream anti-metastatic micro-RNA (mir-200) family expression. The increase in mir-200 consequently downregulated the epithelial mesenchymal transition and lung metastasis [76].

### 3.2. Oncogenic Activity of TETs

In stark contrast to its tumor suppressing functions, TETs could also activate multiple downstream oncogenic signaling pathways by demethylating the epigenome. TET1 expression was demonstrated to promote cell metastasis in colorectal cancer and activation of PI3K oncogenic signaling in TNBC. TET1 expression was shown to correlate with cell migration, cancer stemness tumorigenicity, and poor survivals in epithelial ovarian cancer and in TNBCs [77,78,79].

The oncogenic functional role of TET1 was also described through different interacting partners such as the circular RNA-FECR. FECR1 binds to the promoter of its gene *FLI1* and recruits TET1 to demethylate the promoter and increase its expression. Increased FECR1 enhanced invasiveness of the TNBC cell line and it was present in advanced and metastatic breast cancers. Interestingly, FECR1 downregulated DNMT1 which may cause activation of many pro-tumorigenic factors [80].

In another study, knock-out of TET2 decreased MCF7 (ER^+^) cell proliferation but did not affect the proliferation of the ER-negative cell line [81]. Moreover, it was shown that loss of TET2 in MCF7 cells increased DNA methylation at enhancer regions and consequently decreased the recruitment of ER alpha in response to estradiol treatment and attenuated the estrogen response. Interestingly, it was also shown that TET2 is a direct target of ER alpha and ER antagonist tamoxifen treatment decreased TET2 mRNA and protein levels. This is also suggestive of the possibility that this TET2–ER axis can be important in development of tamoxifen resistance however this needs further investigation. Furthermore, estradiol-regulated TET2 expression was dependent on the recruitment of MLL3 at the active enhancers of the *TET2* gene only in ER-positive cells. Moreover, the MLL3 and TET2 correlation was found to be greater and more significant in breast cancer patient samples with ER-positive than in ER-negative tumors [81].

### 3.3. Oncogenic Activity of TET1 in TNBC

TNBC tumors have a distinct epigenome with lower gains of promoter CGI hypermethylation and have marked hypomethylation at non-CGI compared to all other subtypes and normal breast controls. Thus, TNBCs provide a unique opportunity to investigate the mechanistic basis of hypomethylation and potentially discover therapeutic intervention for this poor prognosis phenotype.

Could this hypomethylated phenotype specifically in TNBC be explained by differential expression levels of DNMT or TET enzymes in breast cancer subtypes? To answer this question, a recent study investigated the TCGA RNA-seq data for normal breast and breast cancer subtypes [71]. It was found that DNMTs were slightly elevated in all breast cancer types compared to normal tissue and therefore they were dismissed as a subtype-specific epigenetic modifier. On the other hand, TET1 was significantly repressed in hormone-receptor-positive cancers, and dramatically overexpressed in TNBCs while TET2 was downregulated in both hormone-receptor-positive breast cancers and TNBCs and TET3 was elevated in all subtypes. These data suggested that TET1 may play an important role in breast cancer specifically in TNBC methylation phenotype and this was confirmed in two independent datasets. Furthermore, it was shown that the levels of TET1 correlated with TET1-mediated DNA hypomethylation in TNBC. Interestingly, a couple of patients with hormone-receptor-positive breast cancer with high levels of TET1 also showed TET1-mediated hypomethylation. These findings confirmed the relationship between TET1 levels and DNA hypomethylation, regardless of cancer subtype. Nevertheless, since TET1 is overexpressed in about 40% of TNBCs compared to only 4% of the other subtypes, TET1-mediated hypomethylation is predominant in TNBCs [71].

Consistent with other studies, in this study, it was also evident that not all TNBCs are the same. Putative TET1 target genes were identified based on the correlation between TET1 expression levels and DNA methylation values [71]. These target sites were enriched for CGI shores and clustered TNBC patients into two groups. One cluster had higher TET1 level and was more hypomethylated and a second group of TNBC patients with lower TET1 and less hypomethylation. These TET1-mediated hypomethylated sites were also enriched in cancer related pathways including but not limited to PI3K/mTOR pathway, Hippo signaling and others. Therefore, TET1 potentially could activate oncogenic signaling in TNBC and as such can be targeted therapeutically. PI3K pathway which regulates cell proliferation, survival and migration is very important in breast cancer. Forty percent of hormone-receptor-positive cancers have activating mutations in PI3K, but this pathway was shown to be more active in TNBC based on gene expression and proteomics data [10]. This study however highlighted an important potential regulatory mechanism of this pathway. Cluster of patients with high TET1 had no mutations in PI3K or PTEN whereas 21% of the cluster with low TET1 have mutations in the pathway. Therefore, activating mutations in PI3K and TET1-mediated hypomethylation are distinct ways of activating the same oncogenic signaling pathway [71].

Furthermore, *TET1* knock-out (KO) experiments in 2 TNBC cell lines (MDA-MB-231 and Hs578T) using CRISPR/cas9 approach, support the oncogenic potential of this enzyme [71]. Single clones of *TET1* KO cell lines were studied and loss of TET1 resulted in reduced cell migration and proliferation. Transcriptomic analysis of these KO cell lines, revealed that TET1-mediated hypomethylation of oncogenic pathways such as the PI3K pathway were downregulated. These data support TET1/PI3K/mTOR as an important therapeutic target in a subset of TNBC patients with high TET1 levels. Besides, by targeting the oncogenic signaling pathway downstream of TET1, there are potentially additional therapeutic interventions. This subpopulation of TNBCs also had suppressed immune system pathway genes and upon loss of *TET1* by CRISPR/cas9, these genes were upregulated. It remains to be determined whether those TNBC patients with high TET1 levels, activated PI3K signaling pathway, and low immune response pathway genes could benefit from treatments targeting TET1 and PI3K immune activators alone or in combination. In the clinic, it is well-known that the basal-like immune-suppressed (BLIS) subgroup of TNBC patients have the worst treatment response and prognosis [82]. Whether TET1/PI3K and immune oncology could offer therapeutic potential for the BLIS subgroup remains to be determined. In contrast, the TNBCs with low TET1 have upregulated immune pathway genes and therefore could be sensitive to immune checkpoint inhibitors. Interestingly, the anti-correlation between TET1 levels and immune markers were corroborated from an independent study. In this study, it was shown that the basal subtype of breast cancer with low TET1 levels has a high expression of immune markers and immune cell infiltration. The study also showed that the p65 component of the major immune regulator NF-kB binds to the TET1 promoter and represses its expression [83]. Though this study did not explicitly indicate it, their findings suggest that these immune-active and low-TET1-expressing cancers represent a separate group of TNBCs such as the basal-like immune active (BLIA). Interestingly this anti-correlation between TET1 and immune regulators was also shown in other cancer types such as melanoma and lung and thyroid cancers. However, whether the relationship between TET1 and immune markers in TNBC is correlative or causative remains to be determined in future investigations. Therefore, together these findings first support the fact that in certain TNBCs when TET1 is high, immune response genes are low, and in other basal-like breast cancers, TET1 is low possibly because of immune system modulating the cancer cell epigenetics. Second, these studies highlight an important paradigm of the heterogeneity of TNBCs and the need to personalize the therapeutic approach.

In another study that supports the oncogenic role of TET1, it was shown that TET1-mediated hypomethylation upregulated gene expressions that drove self-renewal and expansion of cancer stem cells in TNBC. This TET1-mediated oncogenic function is an alternate mechanism that was shown to be due to the downregulation of the catalase enzyme which increases the hydrogen peroxide levels, or be due to exogenous routes such as systemic inflammation and oxidative stress within the context of obesity in TNBC [77].

### 3.4. TET1^ALT^ Is a Novel Isoform of TET1

TET1 contains a CXXC domain that targets it to unmethylated CGIs. Previously, it has been shown that overexpression of TET1 in HEK293T cells does not induce demethylation in most CpG sites surveyed instead it was suggested to function as a guardian protecting the unmethylated CpG islands from methylation [54,84]. However, in contrast to its guardian role, TET1-mediated hypomethylated sites in TNBCs were enriched for non-CpG island DNA, including enhancers, shores, and gene bodies. Thus, it was proposed that TET1 in TNBC acts more like TET2 which lacks the CXXC domain and targets non-CGI DNA [61]. Indeed, this paradox led to the investigation and discovery of an alternate TET1 isoform (TET1^ALT^) that lacks the CXXC domain but retains its catalytic activity. In fact, the canonical TET1 protein (TET1^FL^) is the only isoform expressed in embryonic stem cells, while the TET1^ALT^ isoform is predominantly expressed in adult cells and is overexpressed in cancers such as breast, uterine, ovarian, AML and glioblastoma. Furthermore, it was also shown that full-length TET1 (TET1e) and the short isoform (TET1s) were differentially expressed in mouse embryonic stem cells and somatic cells, respectively [85].

TET1^FL^ is a large protein (2136 amino acids) that is transcribed from a typical CGI-containing promoter [61]. On the other hand, an unmethylated CpG site was found in intron 2 just upstream of exon 3 that was conserved among primates and placental mammals. This site was not only unmethylated but also enriched for H3K4me3 mark, POLII, H3K27Ac, and was aligned with the start of two expressed sequence tags, suggestive of an alternate TET1 promoter transcribing an alternate isoform of the TET1 protein (TET1^ALT^) that lacks the CXXC domain (exon 2). Indeed, when this alternate region was cloned into the luciferase vector, promoter activity was detected. Furthermore, analysis of the raw RNAseq data for breast cancer from the TCGA database revealed expression from this alternate promoter in many TNBC cases suggesting that TET1 overexpression in TNBC cases is partly driven by activation of TET1^ALT^ promoter. TET1^ALT^ protein was also as efficient as the TET1^FL^ protein in generating 5hmC product in overexpression studies [61].

Interestingly, overexpression of the TET1^FL^ and TET1^ALT^ in HEK293T cells resulted in demethylation of distinct CpG sites. Furthermore, RNAseq analysis of the cells overexpressing either the full length or the alternate isoform revealed that TET1^FL^ and TET1^ALT^ cluster separately and there was moderate overlap between the gene expression targets [61]. These data illustrate that these alternate TET1 proteins have different gene expression and methylation targets and possibly distinct physiological roles and hence would require further investigation in the setting of TNBCs.

### 3.5. Clinical Implications of TET1 in TNBCs

While research studies have focused on understanding the roles of aberrant DNA methylation events in driving breast carcinogenesis, as prognostic tools and as tools to distinguish breast cancer subtypes, both levels, and alternate isoforms of TET1 have come to shed light on the mechanism of hypomethylation in one of the most hypomethylated tumor types. Moreover, it was recently shown that TET1 has a prognostic potential because TNBCs with high levels of TET1 (the most hypomethylated tumors) had worse overall survival compared to TNBC with low levels of TET1. In fact, high levels of TET1 were associated with worse overall survival in uterine and ovarian cancers as well [61].

In a previous study DNA methylation, particularly hypermethylated differentially methylated regions, were used to stratify TNBCs into survival groups. In contrast to the TET1-associated survival outcome, in this study [60], the TNBCs with the most hypomethylated had better overall survival compared to intermediate and high levels of methylation. However, this study was designed to identify differentially hypermethylated regions in tumors compared to the normal control and used those sites to stratify the TNBC samples into survival groups [60]. Therefore, the hypomethylated samples that the authors refer to are in fact the least hypermethylated because the normal samples at baseline had low levels of methylation and therefore could not further hypomethylate. Thus, active hypomethylation in cancer (by, for example, TET enzyme) as seen at sites that are normally methylated at baseline is not to be confused with tumors with fewer gains of methylation compared to unmethylated normal samples. Additionally, this study [60] used MBDCap-Seq in their discovery cohort that is biased towards hypermethylated regions, has low base-pair resolution and methylation of non-CpG and areas with less dense 5mC are not well covered by this method. Additionally, when associating changes in methylation levels with survival outcomes, it is important to remember that DNA methylation changes are context-dependent.

However, based on the current literature, the clinical translation of the roles of TETs, even of TET1, particularly in TNBCs is premature. Indeed, current clinical trials are, so far, designed from the mindset that TET enzymes are tumor suppressors and need to be reactivated or their levels should be elevated. These clinical trials (NCT03397173, NCT03433781, and NCT03999723) in myelodysplastic syndrome, acute myeloid leukemia, and others have been focused on investigating the efficacy and safety of oral vitamin C, an activator of TET enzymes, in combination with DNMT inhibitors to potentiate the hypomethylating effect in patients with TET2 mutant cancers. However, in the future and with more thorough research on a larger population, it could be important to investigate whether TNBC patients with high TET1 should avoid vitamin C or not. On the other hand, two groups report on discovery of inhibitors of TET enzymes and therefore reflect the need to inhibit the oncogenic roles of TETs. In one of these studies [86], the cytosine-based TET enzyme is, however, reported to inhibit the enzyme’s activity in mid-micromolar concentrations. In another study [87], authors report on discovering a small molecule inhibitor of TET enzyme’s catalytic activity that would facilitate the differentiation between the catalytic and non-catalytic activities of these enzymes. Though these reports hint at preliminary attempts to discover TET inhibitors, there is much work to be done to improve target specificity, off-target effects, and assay development for the outcome measurements, etc., and therefore they are still far from clinical application.

**Figure 2:**
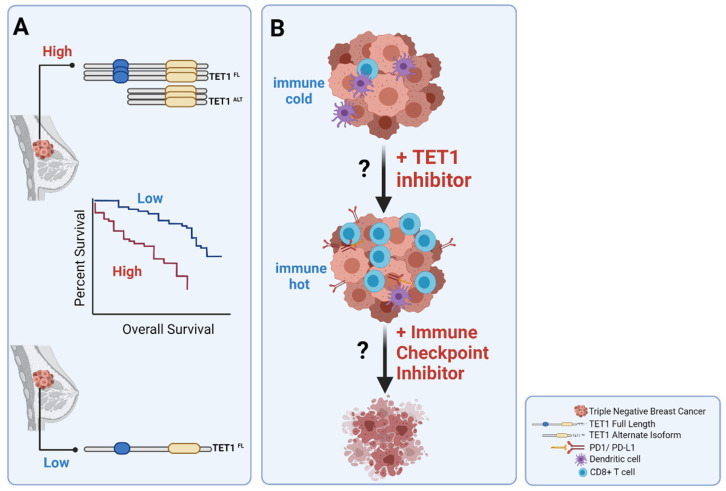
Proposed model for therapeutic potential of TET1 in TNBC. (**A**) TNBC tumors can be subdivided into TET1-low and TET1-high (full length and/or alternate isoform) groups. Patients with high TET1 tumors have worse overall survival compared to patients with low TET1 tumors as indicated in the Kaplan–Meier curves. (**B**) Tumors with high TET1 are characterized by low immune response genes and are immune-cold. The model suggests that inhibition of TET1 will result in activation of immune pathway genes that could potentially respond to immune checkpoint inhibitors and improve overall survival.

Furthermore, although currently there is no evidence that TET1 levels in TNBC could be used as a biomarker to predict response to immunotherapy, there is emerging evidence that TET1 mutant samples in several cancers were more immunogenic and showed better responses to immune checkpoint inhibitors [88]. Similarly, in the future, one might envision using TET1 levels as a prescreening tool to stratify TNBC patients for immune therapy as highlighted in the proposed model (Figure 2). Based on the published data, our model hypothesizes that TNBC patients with low TET1 have upregulated immune mediators and could be sensitive to immune checkpoint inhibitors while TNBC patients with high TET1 have decreased expression of immune-pathway genes. Therefore, the high TET1 TNBC subgroups based on the hypothesized model, could benefit from inhibition of TET1 in combination with immune checkpoint inhibitors.

## 4. Conclusions and Future Directions

In this review, we highlight the role of DNA demethylases in TNBCs. We presented evidence based on the literature that TET enzymes can act as oncogene and tumor suppressors depending on cellular context in breast cancer. The opposing roles of TET enzymes add a layer of complexity in breast cancer, making them more interesting and the target for either inhibition or activation, and the subject of future studies to parse out their roles. Based on the literature review presented in this manuscript, one plausible explanation for the opposing roles of TET is the demethylation and direct or indirect activation of downstream tumor-suppressor genes or oncogenes. This in turn highlights the reliance of TET enzymes, which lack sequence specificity, on interacting partners to be recruited to targeted sites. Therefore, depending on the availability of these various interacting partners, TETs can be targeted to different regions and play different roles. More importantly, the controversial roles of TET1 may be isoform-specific; whereby full-length TET suppresses tumors by protecting CpG islands from gaining methylation while the alternate isoform of TET functions as an oncogene by promoting demethylation of targeted sites based on tissue-specific interacting partners. In fact, the contradictory roles of the TET enzyme as a tumor suppressor and as an oncogene have also been observed in other epigenetic regulators such as DNMT3A and EZH2 [35,89,90]. Importantly, one could find examples of hypomethylation driving leukemias (DNMT3A mutations) [62] as well as of hypermethylation (IDH mutations) [91] driving the leukemia. This phenomenon then aligns with the Goldilocks principle, where too much or too little of methylation/hydroxymethylation is deleterious and an optimal amount is desired for proper epigenetic regulation.

## Figures and Tables

**Table 1:** DNMT inhibitors.

Chemical Name	Generic Name	Mechanism	Drug Status
5-Azacytidine	Azacitidine	Cytosine analog	FDA- and EMA-approved for treatment of MDS
5-Aza-2-deoxycytidine	Decitabine	Cytosine analog	FDA-approved for treatment of MDS and EMA-approved for treatment of AML
SGI-110	Guadecitabine	Cytosine analog	Phase III clinical trial in AML

FDA, US Food and Drug Administration; EMA, European Medicines Agency; MDS, myelodysplastic syndrome; AML, acute myeloid leukemia.

## Data Availability

Data sharing not applicable.
